# Interface geometry governs probe insertion mechanics and electrode deviation in layered brain-mimicking phantoms for deep brain stimulation

**DOI:** 10.1007/s10237-026-02122-1

**Published:** 2026-09-16

**Authors:** Siyu Chen, Chengyuan Wu, Ethan Anders, Rungun Nathan, Olivia M. Bailey, Qifu Wang, Curtis L. Johnson, Qianhong Wu

**Affiliations:** 1 Cellular Biomechanics and Sports Science Laboratory, Villanova University, Villanova, PA USA; 2 Department of Mechanical Engineering, Villanova University, Villanova, PA USA; 3 Department of Neurological Surgery, Thomas Jefferson University, Philadelphia, PA USA; 4 Department of Radiology, Jefferson Magnetic Resonance Imaging Center, Thomas Jefferson University, Philadelphia, PA USA; 5 Pennsylvania State University Berks, Reading, PA USA; 6 Department of Biomedical Engineering, University of Delaware, Newark, DE USA

**Keywords:** Deep brain stimulation, Electrode deviation, Probe insertion, Tissue interface, Magnetic resonance elastography, Biomechanics

## Abstract

Parkinson’s disease (PD) is a progressive neurological disorder often treated with Deep Brain Stimulation (DBS), a surgical procedure that involves implanting electrodes to improve motor function. Accurate electrode placement is critical for treatment efficacy but can be influenced by tissue deformation, insertion forces, and the mechanical properties of brain tissue. In this study, we investigated the role of layer interface angle as a controlled biomechanical factor affecting insertion forces and electrode deviation. A two-layer agar gel phantom was constructed to produce a prescribed stiffness transition between a lower-stiffness and a higher-stiffness gel layer, and its small-amplitude dynamic shear-stiffness contrast was characterized using magnetic resonance elastography (MRE) at 100 Hz. A stage-wise analytical model was formulated to describe the Z-direction axial force–depth response ${F}_{z}\left(z\right)$ during indentation, fracture, relaxation, and frictional sliding, and a hybrid physics-guided residual neural network (PGNN) was explored as a secondary residual-correction approach for the remaining axial-force model discrepancies. The lateral interface-force change ${F}_{iy}$ was obtained directly from the measured Y-direction force signal, while electrode deviation was quantified in a separate series of phantom experiments. Insertion experiments at interface angles of 0°, 15°, 30°, 45°, 60°, and 75° demonstrated that the lateral (Y-direction) interface force increased from 0.886 mN at 0° to 7.72 mN at 75°. For the deviation analysis, the mean value of the 0° group was defined as the zero reference; relative to this baseline, the largest group mean deviation was 0.0529 mm at 75°. These findings highlight the role of interface geometry in probe trajectory stability and provide a mechanistic baseline for future studies of probe insertion in two-layer media.

**Supplementary Information:**

The online version contains supplementary material available at https://doi.org/10.1007/s10237-026-02122-1.

## Introduction

Parkinson's disease (PD) is a progressive neurodegenerative disorder that significantly affects the quality of life of millions of individuals worldwide. It is characterized by motor symptoms such as tremor, rigidity, and bradykinesia, which worsen over time and impose a substantial burden on patients, caregivers, and healthcare systems. Recent estimates indicate that nearly 90,000 people are diagnosed with PD annually in the USA, with the global burden of the disease resulting in 5.8 million disability-adjusted life years (DALYs) in 2019, marking an 81% increase since 2000 (Ray Dorsey et al. 2018; Marras et al. 2018). While pharmacological therapies, such as levodopa, are effective in managing early-stage symptoms, their efficacy diminishes as the disease progresses, leaving patients with unmet clinical needs (Beckers et al. 2022).

**Fig. 1:**
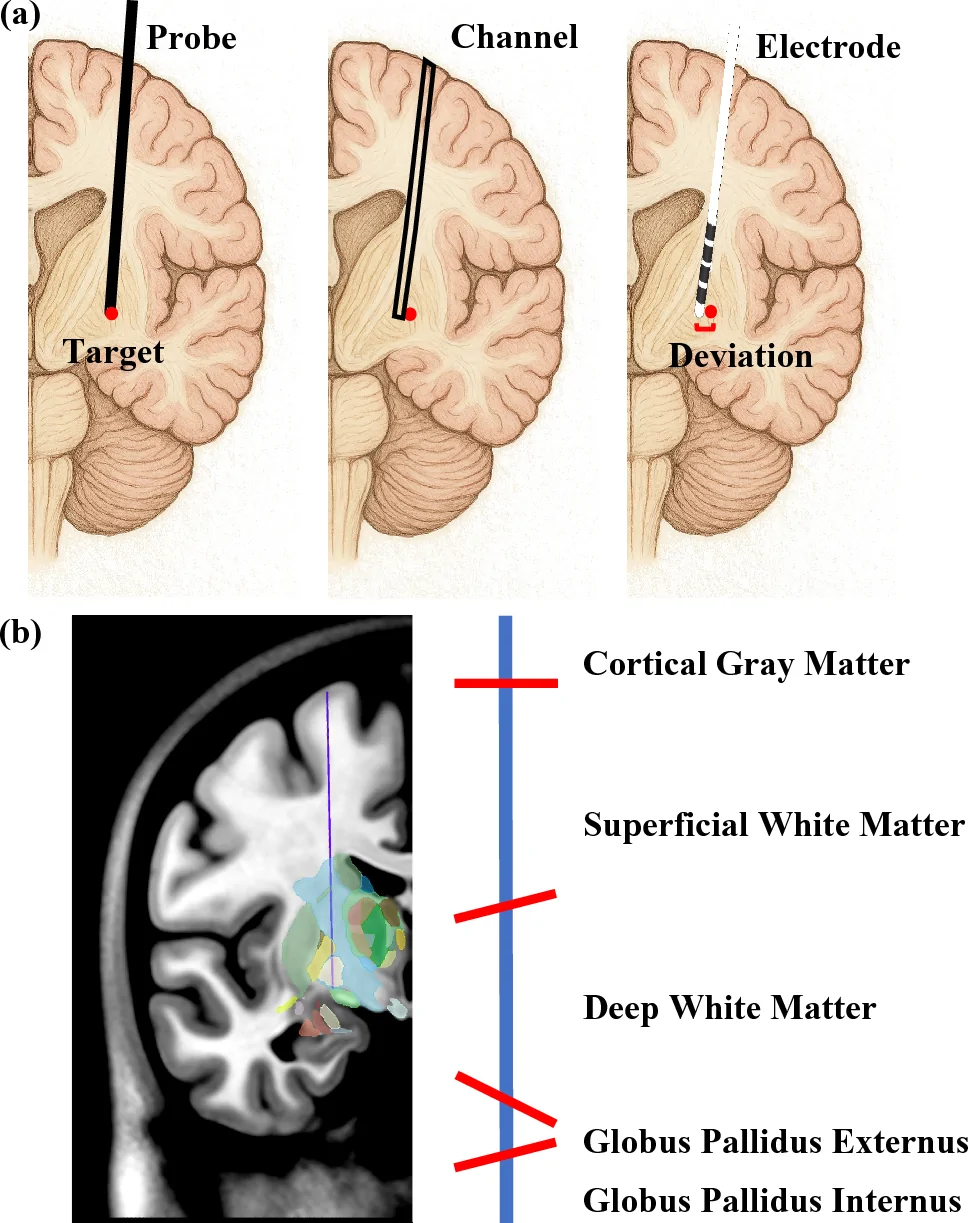
**a** Steps of a typical DBS procedure. Left: A probe is first inserted into the brain, passing through multiple structures to reach the target. Middle: The probe is then withdrawn, creating a channel. The shape, size, and location of this channel may be influenced by local tissue properties, and deflection can occur especially at interfaces. Right: The DBS electrode is inserted along the preformed channel toward the target region. However, deformation of the channel may lead the electrode to deviate from the target. **b** The trajectory of the electrode reaching the DBS target passes through multiple interfaces. Taking the Globus Pallidus Internus (GPi) as an example, the trajectory intersects with an average of four distinct tissue interfaces, each with different angles

Deep Brain Stimulation (DBS) has served as a transformative surgical intervention for advanced PD patients. By implanting electrodes into specific brain regions to modulate neural activity, DBS provides significant improvements in motor symptoms and overall quality of life (Miocinovic et al. 2013). One commonly used technique involves inserting a rigid cannula along the planned trajectory, with or without the use of microelectrode recordings for physiological confirmation. Another method employs a radiofrequency (RF) probe with dynamic impedance monitoring to establish a low-resistance channel, allowing direct electrode insertion after probe withdrawal without a cannula (Foltynie et al. 2011) (Fayed et al. 2024) (Ziechmann et al. 2026), as illustrated in Fig. 1a. Electrode placement accuracy and deviation have been shown to be comparable between these two approaches (Rajabian et al. 2023). However, the success of DBS is highly dependent on the accuracy of electrode placement**,** and enhanced targeting strategies have been a key driver in the evolution of DBS applications (Burchiel et al. 2015). Suboptimal electrode placement can compromise therapeutic efficacy and increase the risk of adverse effects, such as dysarthria and motor deficits (Kluin et al. 2022).

Electrode placement during DBS is influenced by several factors, including anatomical variations, accuracy of the stereotactic system, and intraoperative factors such as global brain shift (Li et al. 2016; Ko et al. 2017). Real-time assessment of electrode location during surgery has been recognized as a key technical challenge (Vissani et al. 2020). Biomechanical factors could also influence electrode placement accuracy. Electrode–tissue interactions, including tissue deformation and probe–tissue interaction, have been shown to significantly contribute to deviations from the intended electrode trajectory (Jiang et al. 2014; Chapelle et al. 2021). During the DBS procedure, the electrode must traverse multiple tissue structures and their interfaces, as illustrated in Fig. 1b. These tissues exhibit distinct biomechanical properties, which can introduce mechanical asymmetry during insertion and contribute to systematic deviations in electrode trajectory. As a probe is inserted or removed, the brain’s viscoelastic properties, anisotropy, and material heterogeneity may generate resistance and reaction forces that impact insertion accuracy. Following the extraction of the probe, the electrode is inserted along the channel created by the probe to reach the planned target. We hypothesize that electrode deviation from the target is influenced largely by biomechanical factors present during probe or cannula insertion, and that these effects can be isolated and quantified using controlled phantom experiments. These deviations may be particularly pronounced at tissue interfaces where mechanical properties differ, such as the boundary between gray and white matter (Budday et al. 2015).

Recent advancements in DBS technology, such as directional leads, adaptive stimulation systems, and advanced imaging techniques, have improved clinical outcomes by increasing tolerance to electrode deviations (Ramirez-Zamora et al. 2018). However, a gap remains in understanding the biomechanical interactions that occur during electrode insertion. Studies have suggested that the probe–tissue interaction and interface angle could influence electrode trajectory (Siyu Chen et al. 2024); yet these factors are not well characterized from a mechanics perspective. In a clinical study of 21 DBS electrode trajectories, the angle between the planned trajectory and the normal to an MRE-defined stiffness interface was significantly correlated with target deviation, with larger angles associated with greater deviations (*r*=0.57, *p*=0.007) (Wu et al. 2025). This finding provides clinical evidence of an association between interface geometry and electrode deviation, while the underlying mechanics remain inadequately characterized. The present study, therefore, isolates interface angle under controlled phantom conditions and addresses three core questions:

1. What mechanical processes characterize the axial force–depth response during probe insertion,
2. How interface angle affects the directly measured lateral interface-force change ${F}_{iy}$, and
3. How interface angle is associated with electrode deviation measured in a separate experimental series.

To address these objectives, we constructed a two-layer agar gel phantom with a prescribed contrast between lower- and higher-stiffness layers, enabling controlled and systematic investigation of interface-driven biomechanical responses. This simplified experimental framework is designed to isolate fundamental interface-driven mechanical effects, rather than replicate the full complexity of in vivo brain tissue. A stage-wise analytical force model was formulated to characterize the measured Z-direction axial force–depth response ${F}_{z}\left(z\right)$ during indentation, fracture, viscoelastic relaxation, and frictional sliding, and a hybrid physics-guided residual neural network (PGNN) was incorporated as a residual correction to this axial-force model**.** The lateral force ${{\boldsymbol{F}}}_{{\boldsymbol{y}}}$ and the derived interface-force change ${F}_{iy}$ were quantified directly from the force-transducer measurements across the prescribed interface angles. Electrode deviation was quantified in a separate series of phantom experiments using high-speed imaging. This combined design enables the axial-force response, lateral interface force, and electrode deviation to be evaluated as complementary outcomes with interface angle as the common controlled variable. Together, these experimental and modeling approaches allow us to characterize axial insertion mechanics and determine the angle-dependent trends in lateral force and electrode deviation under controlled phantom conditions, providing insights that advance the mechanistic understanding of probe–tissue interactions in two-layer media and may inform future studies of insertion mechanics in more complex tissue environments.

## Materials and methods

We first investigated the mechanical properties of agar gel at different concentrations using Magnetic Resonance Elastography (MRE). A custom-designed system was constructed in which a probe, driven by a motor, was inserted into a two-layer gel with interface angles set at 0°, 15°, 30°, 45°, 60°, and 75°. Reaction forces on the probe were measured. After the probe was removed, a DBS electrode was inserted. High-speed imaging captured the probe insertion, removal, and electrode placement processes, enabling quantification of electrode deviation from the intended trajectory.

### Experimental materials and MRE test

Agar gel (Sigma-Aldrich 05039) was selected as a transparent and mechanically tunable phantom material that facilitates real-time visualization during the experiments. The previous studies have demonstrated the utility of agar-based gels in optically accessible brain phantom experiments (Albor-Ramírez et al. 2023) and in controlled investigations of brain tissue injury patterns (Falland-Cheung et al. 2016). To establish a prescribed stiffness contrast motivated by the mechanical heterogeneity among brain regions (Hiscox et al. 2020), 0.6% w/w agar gel was used as the lower-stiffness outer layer and 0.7% w/w agar gel as the higher-stiffness inner layer. These designations refer to the relative stiffness of the two layers within the phantom and do not imply full mechanical equivalence to gray matter, white matter, or deep brain nuclei. Preparation followed a standardized protocol: Agar gel and water were weighed by mass fraction, mixed, heated to 90–95 °C, poured into containers, and refrigerated for 24 h to solidify (McIlvain et al. 2019).

To characterize the small-amplitude, frequency-specific dynamic response of the gel phantoms, MRE was employed to quantify stiffness and damping ratio by imaging the displacement fields induced by harmonic vibrations (Muthupillai et al. 1995). MRE operates by generating shear waves within the material, acquiring MR images of wave propagation, and processing these images to produce quantitative stiffness maps known as elastograms. This technique has demonstrated excellent repeatability and interobserver consistency (Murphy et al. 2019), and has shown promising applicability in a variety of contexts, including brain imaging (Nanjappa and Kolipaka 2021). Vibrations at 100 Hz were applied to agar gel samples using a pneumatic actuator system (Resoundant; Rochester, MN). MRE data were acquired on a Siemens 3T Prisma MRI scanner using an EPI sequence with 2.5 mm isotropic spatial resolution (Chaze et al. 2019). To ensure the consistency and stability of the agar gel phantoms used for MRE, all samples were prepared on the same day by a single experimenter following a standardized protocol (McIlvain et al. 2019). Each sample was created using 1000 mL of water and cast into a disposable square plastic container (dimensions: 156 mm × 156 mm × 86 mm; volume: 1180 mL). Each phantom was allowed to solidify within its container for 24–48 h and was scanned in situ during MRE acquisition. These 100-Hz MRE measurements were used to characterize the dynamic stiffness contrast between the gel formulations and were not used to infer their finite deformation, rupture, or frictional behavior during probe insertion.

### Experimental setup

#### Force measurement experiment

**Fig. 2:**
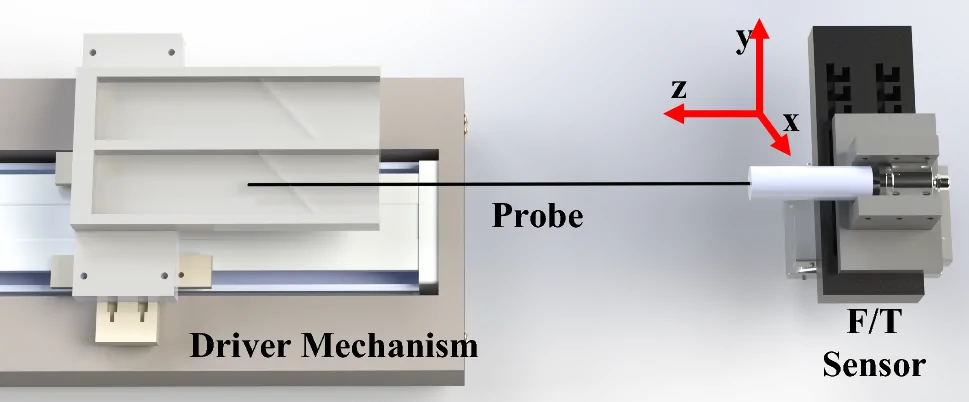
Schematic diagram of force measurement experiment

Figure 2 provides the schematic of the force measurement setup. To minimize noise caused by vibrations from the driving mechanism, the force sensor was directly attached to the fixed probe. The two-layer agar gel was mounted on a platform that could be set in motion by the driving mechanism, allowing the insertion and removal of the probe from the agar gel. The maximum insertion depth for the force measurement experiments was 80 mm, and the insertion speed was 19.3 mm/s. Six experiments were performed for each interface angle (total of 36 experiments). In this study, we used the same 1.8-mm-diameter probe (Cosman G4, Boston Scientific) employed in DBS procedures at Thomas Jefferson Hospital (Fayed et al. 2024; Ziechmann et al. 2026). The probe, made of Nitinol (nickel–titanium alloy), has a 4 mm exposed tip and a total length of 29 cm and a Young’s modulus around 74.21 GPa (Massey et al. 2021), which is several orders of magnitude higher than that of the agar gel. Therefore, the probe can be reasonably regarded as a rigid body during its interaction with the gel. Force measurements were performed using an IP65 Nano17 transducer (ATI Industrial Automation, Apex, NC), which is capable of capturing forces and torques along three axes with a resolution as fine as 0.0001 N. Data acquisition and processing were conducted using NI-9205 and NI cDAQ-9174 system (National Instruments, Austin, TX) with a sampling rate of 3000 Hz for each input. A custom LabVIEW program synchronized data collection from multiple signal sources. The transducer directly recorded the Z-direction axial force ${F}_{z}$ and the Y-direction lateral force ${F}_{y}$. The measured ${F}_{z}\left(z\right)$ response was used to identify and evaluate the stage-wise analytical and PGNN-corrected axial-force models, whereas the interface-force change ${F}_{iy}$ was calculated from the measured ${F}_{y}$ signal and analyzed as an experimental outcome. To reduce random electrical noise, the input signal was filtered using a third-order Butterworth low-pass filter with an 80 Hz cutoff frequency.

Force measurements were performed in two sequential stages. First, homogeneous single-layer agar phantoms were tested to identify the layer-specific parameters used in the analytical model. Single-layer phantoms were prepared using 0.6% w/w agar and 0.7% w/w agar, corresponding to the outer and inner layers of the subsequent two-layer configuration. Because these phantoms were mechanically homogeneous, the measured force–displacement response reflected the intrinsic insertion and withdrawal behavior of each individual material without the added complexity of an interface. Controlled insertion and withdrawal tests were performed for each concentration, with six repeated trials conducted per material. The resulting force–displacement curves were used to determine the layer-specific parameters for nonlinear indentation, post-fracture relaxation, cutting resistance, and probe–gel friction, which were then transferred to the two-layer model.

Second, two-layer force measurement experiments were performed to evaluate interface mechanics under different interface angles. In these experiments, the phantom consisted of an outer 0.6% w/w agar layer and an inner 0.7% w/w agar layer, with interface angles set at 0°, 15°, 30°, 45°, 60°, and 75°. The maximum insertion depth was 80 mm, and the insertion speed was 19.3 mm/s. Six experiments were performed for each interface angle, for a total of 36 two-layer force measurements. In contrast with the single-layer tests, these experiments were designed to capture the additional force variations associated with interface crossing, including axial and lateral loading changes that arise from stiffness transition and interface geometry. Together, the single-layer calibration experiments and the two-layer force measurement experiments enabled separation of layer-specific material behavior from interface-driven force asymmetry.

#### Electrode deviation experiment

**Fig. 3:**
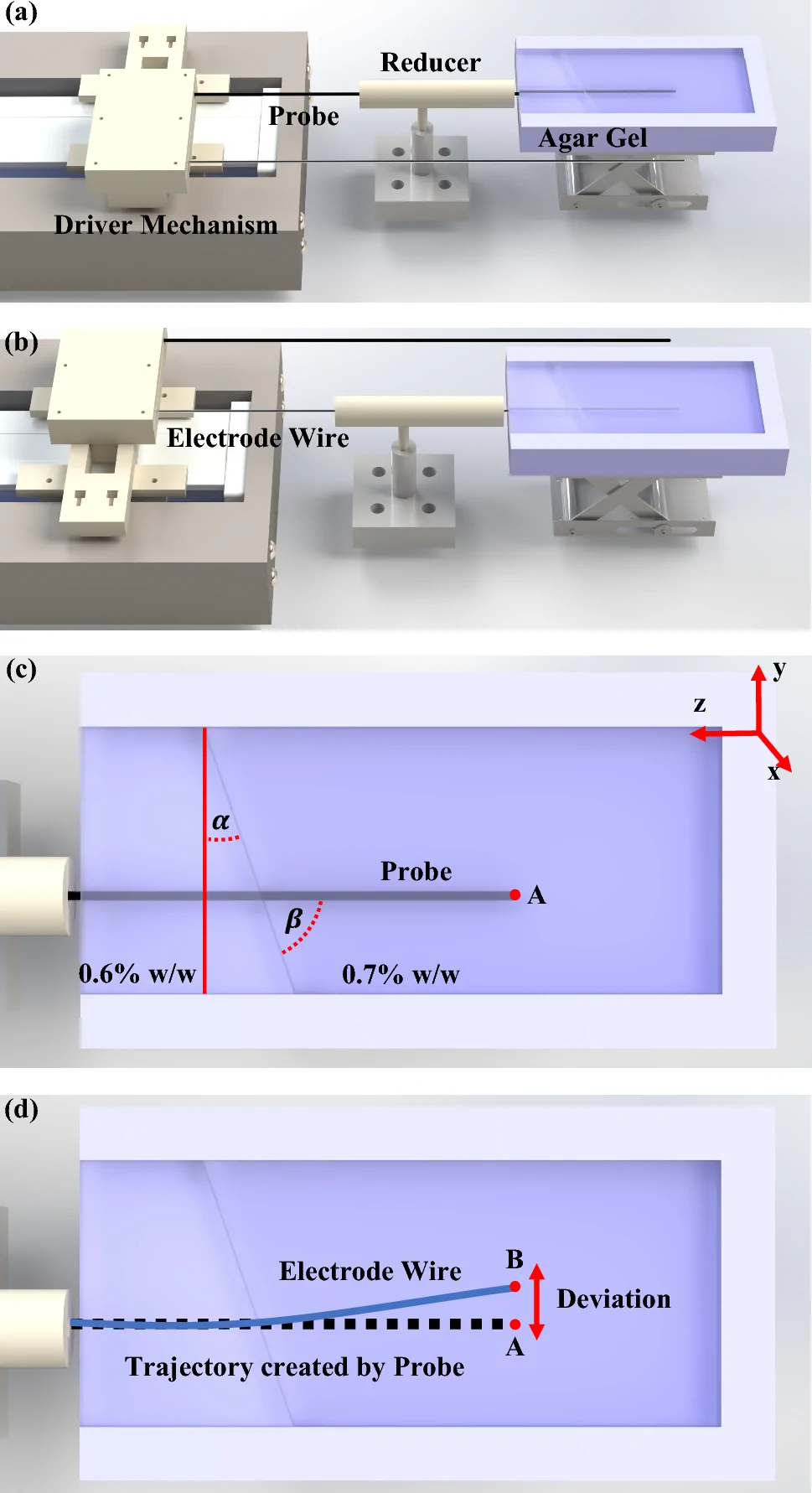
**a** Probe inserted into agar gel **b** After removing the probe, the electrode is inserted into agar gel **c** Schematic diagram of the two-layer agar gel, where ${\boldsymbol{\alpha}}$ is the interface angle between the probe trajectory direction and the interface normal (${\boldsymbol{\alpha}}={0}^{\circ }$ at normal incidence), and ${\boldsymbol{\beta}}$ is the complementary in-plane angle between the probe trajectory and the interface line **d** Schematic diagram of the deviation of the electrode insertion position from the position A. A demonstration video S1 is provided in the supplementary materials

In a separate experimental series, we used the two-layer agar gel phantom to observe and quantify electrode deviation. The force measurement and electrode deviation datasets were acquired using separate phantoms and experimental trials, with interface angle serving as the common controlled variable for group-level comparison. The experimental setup, shown in Fig. 3, includes a linear driving mechanism that enables precise control of probe and electrode speed and displacement, thereby ensuring accurate and reliable insertions. For this experiment, the DBS electrode used was a 40 cm–long, 1.27 mm–diameter 8-channel directional lead (Infinity 6172, Abbott, Plano, TX) (Fayed et al. 2024)(Ziechmann et al. 2026). The distal tip of the electrode consists of four 1.5 mm tall rigid platinum–iridium electrodes spaced 0.5 mm apart on a flexible insulated polycarbonate polyurethane substrate. In addition, a reducer with an inner diameter of 1.8 mm and length of 73 mm was used to maintain a straight trajectory and replicate surgical tools used in DBS implantation surgeries. The probe was first inserted into the agar gel through the reducer and then withdrawn to create a channel. The maximum insertion depth was 80 mm, and the movement speed was 20.0 mm/s. An industrial flare camera (IO Industries, Canada) was positioned above the agar gel to capture the insertion process, with an imaging resolution of 4096 × 3072 pixels and a frame rate of 100 FPS. The slider was then moved to allow the insertion of the electrode wire through the preformed channel, as shown in Fig. 3a, b. Agar gel samples were placed in a custom-designed container featuring side openings to facilitate probe insertion and removal. The samples consisted of two layers with varying interface angles, as illustrated in Fig. 3c. In three-dimensional vector form, the interface angle was defined as $\alpha ={cos}^{-1}(\mid {\boldsymbol{t}}\cdot {\boldsymbol{n}}\mid ),$ where ${\boldsymbol{t}}$ is the unit tangent vector along the probe trajectory, and ${\boldsymbol{n}}$ is the unit normal to the interface. For the planar, coplanar geometry used in the present experiments, this definition is equivalent to the two-dimensional construction shown in Fig. 3c, with $\alpha ={0}^{\circ }$ representing normal incidence. The angle β denotes the complementary in-plane angle between the probe trajectory and the interface line.

A thin acrylic plate was positioned on top of the container, allowing unobstructed visualization while preventing sample deformation. To capture deviations in probe and electrode positions, a high-speed camera was mounted above the agar gel samples. If the position of the probe at target depth is defined as A, and the position of the electrode at target depth is defined as B, then the deviation is defined as the difference in the Y-direction between A and B (Fig. 3d). For subsequent comparisons, the mean measured deviation of the 0° group was defined as zero. The reported group means at 15°–75° therefore represent deviations relative to this zero-referenced baseline. These deviations were experimentally measured for different values of α. Ten independent electrode deviation trials were conducted at each of the six interface angles, yielding 60 deviation trials in total.

**Fig. 4:**
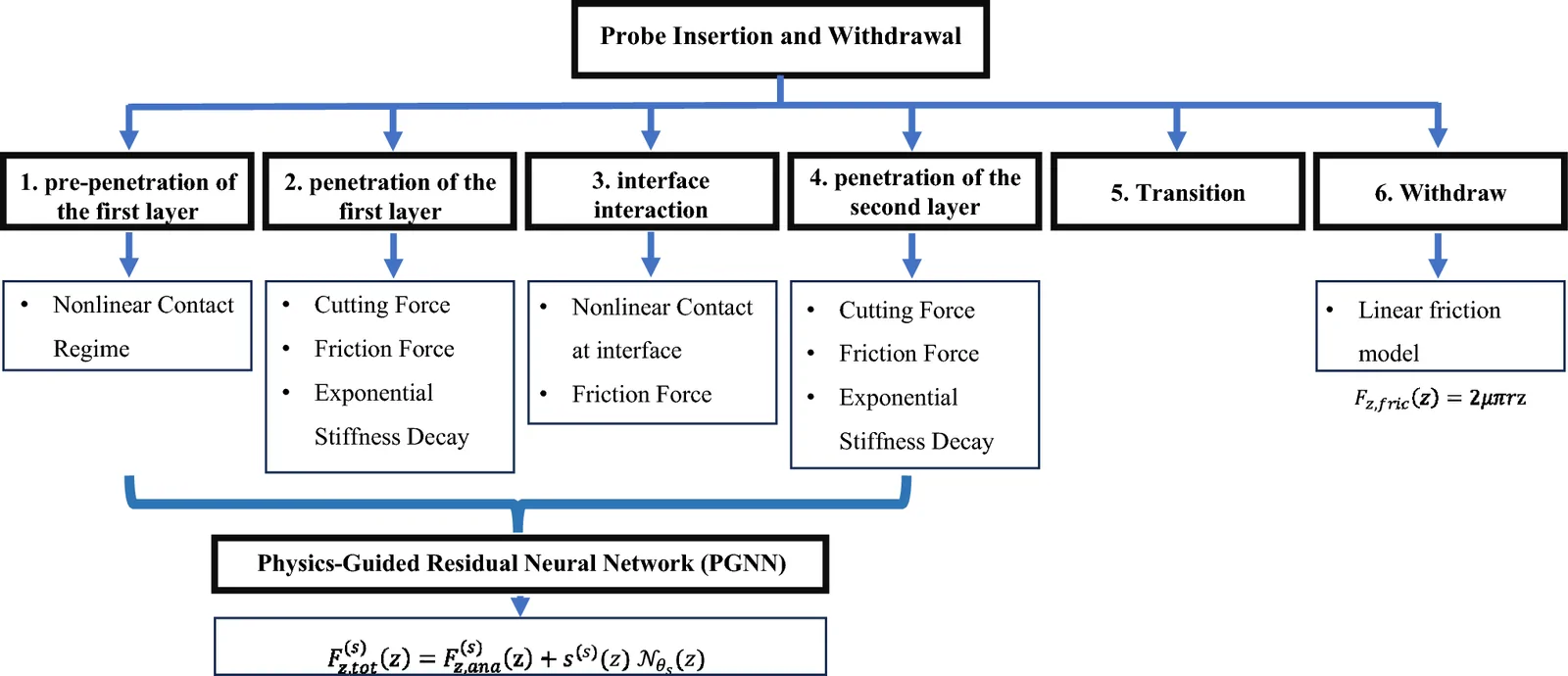
Flowchart of the stage-wise axial-force model for probe insertion and withdrawal. The process is divided into six stages: pre- and post-fracture insertion for each gel layer, a transition phase during motion reversal, and withdrawal dominated by friction. Distinct scalar axial-force relations are applied to each stage according to the dominant interaction mechanisms

#### Statistical analysis

Statistical analysis was performed to quantify the relationships between interface angle and the measured mechanical or geometric response variables. Pearson correlation analysis was used to evaluate the association between interface angle and the Z-axis force change at the interface $\left({F}_{iz}\right)$, the Y-axis force change at the interface $\left({F}_{iy}\right)$, and electrode deviation. In addition, one-way analysis of variance (ANOVA) was used to compare electrode deviation across interface angle groups. Statistical significance was defined as p<0.05. Force and deviation data are reported as mean ± standard deviation unless otherwise stated.

### Stage-wise axial-force model for probe insertion and withdrawal

To characterize the measured Z-direction axial force–depth response ${F}_{z}\left(z\right)$, the probe insertion and withdrawal processes were divided into six distinct stages (Fig. 4): (1) pre-penetration of the first gel layer, characterized by pronounced nonlinearity before full penetration; (2) post-fracture insertion into the first gel layer, dominated by frictional and cutting forces; (3) pre-penetration of the second gel layer, again exhibiting nonlinear characteristics prior to full penetration; (4) post-fracture insertion into the second gel layer, governed by frictional and cutting forces; (5) a transitional phase during which the force direction reverses as motion shifts from insertion to withdrawal; and (6) probe withdrawal, primarily dominated by frictional interactions. Different axial-force relations were applied to each phase according to the dominant interaction mechanisms. The resulting model provides a stage-wise representation of the scalar axial force ${F}_{z}\left(z\right)$**.** The lateral force ${F}_{y}$**,** the derived interface-force change ${F}_{iy}$, and electrode deviation are evaluated as complementary experimental outcomes through the procedures described in Sects. 2.2.1 and 2.2.2.

#### Insertion of the probe into the two-layer gel

Stage 1: pre-penetrating indentation of the outer (first) gel layer

**Fig. 5:**
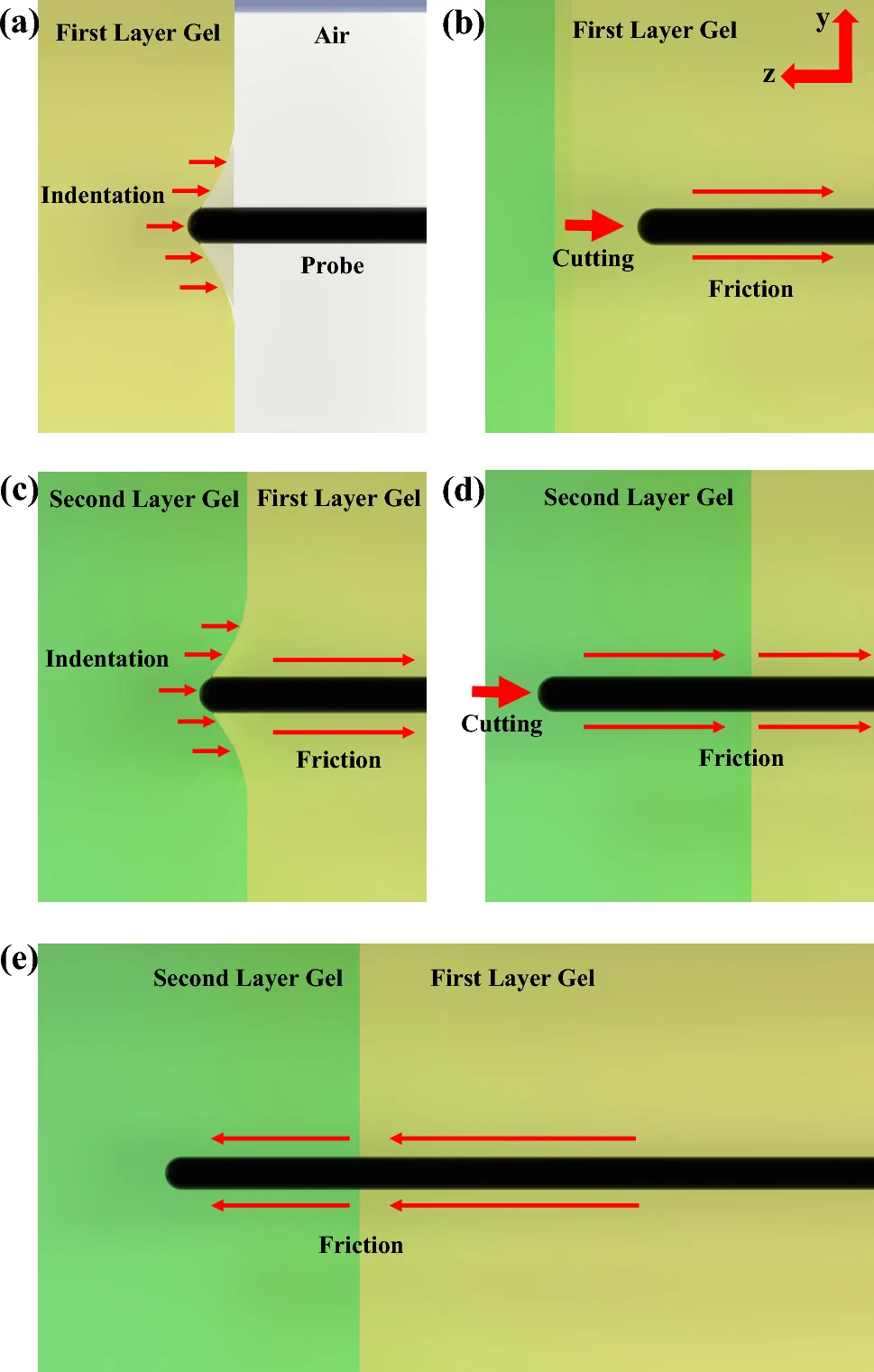
Schematic illustration of the probe–gel interaction stages during insertion and withdrawal in a two-layer agar gel: **a** pre-penetration of the first layer; **b** post-fracture insertion with cutting and friction; **c** pre-penetration of the second layer; **d** post-fracture insertion into the second layer; and **e** withdrawal dominated by interfacial friction

As shown in Fig. 5a, during the pre-penetration stage, the probe tip indents the surface of the first gel layer before fracture occurs. In this stage, the agar gel exhibits pronounced nonlinear deformation behavior, and the reaction force increases rapidly with indentation depth. Such nonlinear stiffening is commonly observed in soft gels and biological tissues and cannot be fully captured by classical linear-elastic contact models such as the Hertz theory (K. L. Johnson 1985). The deviation from the Hertzian prediction mainly arises from large deformation, viscoelasticity, and intrinsic material nonlinearity (Efremov et al. 2015).

To better describe this nonlinear force–displacement relationship, we adopted a Fung-type exponential relation as a compact phenomenological representation of the measured pre-puncture response (Fung 1967; Gefen et al. 2003). The relation was used to capture the observed nonlinearity under the present insertion conditions rather than as a complete constitutive law for brain tissue. The Fung model relates nominal stress σ to strain ε through

1 $$\sigma = \frac{1}{\beta }\left( {e^{\beta k\varepsilon } - 1} \right),$$

where $\varepsilon =\left(z-{z}_{s}\right)/R$ is a dimensionless indentation measure. In the present N–mm implementation, k is an effective stress-scale parameter with units of N/mm^2^, whereas β is an inverse-stress nonlinearity parameter with units of mm^2^/N. Consequently, $\beta k\varepsilon$ is dimensionless, and 1/β has units of stress. For indentation, the z is the indentation depth, and *R* is the probe radius. Assuming the nominal stress is given by $\sigma =F/(\pi {R}^{2})$, the relationship between the measured Z-direction axial force ${F}_{z}$ and indentation depth z can be derived as

2 $${F}_{z,1}\left(z\right)=-\frac{\pi {R}^{2}}{{\beta}_{1}}\left({e}^{\frac{{\beta}_{1}{k}_{1}\left(z-{z}_{s1}\right)}{R}}-1\right),$$

where $R=0.9\hspace{0.17em}mm$ is the probe radius, ${k}_{1}$ and ${\beta}_{1}$ are the effective stress-scale and inverse-stress nonlinearity parameters, respectively, which were obtained by fitting the experimental force–displacement data from single-layer 0.6% agar gel tests, and ${z}_{s1}$ denotes the onset position of this stage. These fitted parameters are specific to the present probe geometry, gel formulation, loading path, and experimental conditions. They were not derived from the MRE measurements and should not be interpreted as constitutive parameters of brain tissue.

Stage 2: Post-penetrating insertion into the outer gel layer

After the gel interface is punctured, as illustrated in Fig. 5b, the total insertion force can be decomposed into three distinct components: (1) an exponentially decaying term that accounts for the stress relaxation immediately following rupture (Abolhassani et al. 2007); (2) a depth-dependent frictional resistance distributed along the probe surface (Okamura et al. 2004); and (3) a constant cutting force acting at the probe tip (Barbé et al. 2007).

Following the framework proposed in the previous soft-tissue insertion studies (Okamura et al. 2004; Barbé et al. 2007; Abolhassani et al. 2007), the cutting force is treated as a constant term, while the frictional resistance is considered proportional to the inserted length and dependent on the interfacial friction properties of the gel. The stress-relaxation behavior is modeled using an exponential decay, reflecting the time- and strain-dependent viscoelastic response of the agar matrix after fracture. Accordingly, the total force during this post-penetration stage is expressed as

3 $${F}_{z,2}\left(z\right)=-\left[{A}_{2}{e}^{-\frac{\left(z-{z}_{0}\right)}{{\tau}_{2}}}+{F}_{z, fric}^{\left(2\right)}\left(z\right)+{C}_{2}\right],$$

where z is the insertion depth, ${z}_{0}$ is the transition point from Stage 1, ${A}_{2}$ is the amplitude of the relaxation term in N, ${\tau}_{2}$ is the relaxation length constant in mm, ${F}_{\mathrm{z},\mathrm{f}\mathrm{r}\mathrm{i}\mathrm{c}}^{\left(2\right)}\left(z\right)$ is the frictional resistance, and ${C}_{2}$ is the constant cutting-force term in N. Because the insertion speed was fixed, depth was used as a surrogate for time in the exponential relaxation term; ${\tau}_{2}$ is, therefore, a relaxation length constant, and the corresponding effective relaxation time is ${\tau}_{2}/v$.

Continuity between the pre- and post-penetration stages is enforced at the transition point $({z}_{0}={z}_{e1})$ through

4 $${A}_{2}={-F}_{z,1}\left({z}_{0}\right)-{F}_{z,fric}^{\left(2\right)}\left({z}_{0}\right)-{C}_{2},$$

where ${z}_{e1}$ denotes the end position of Stage 1, corresponding to the fracture point of the outer layer, ensuring a smooth force transition across the fracture interface. The frictional component in this regime is modeled as a linearly increasing function of insertion depth,

5 $${F}_{z,fric}^{\left(2\right)}\left(z\right)=2\pi R{\mu}_{2}z,$$

where ${\mu}_{2}$ is the effective interfacial frictional shear-stress parameter (N/mm^2^) identified from the single-layer 0.6% agar experiments.

Stage 3: Pre-penetrating indentation of inner gel layer

As the probe proceeds into the second gel layer, it encounters a new pre-penetration phase before fracturing the inner interface, as illustrated in Fig. 5c. Similar to Stage 1, this stage is characterized by pronounced nonlinear stiffening as the gel resists indentation prior to rupture. However, due to the pre-compression and confinement effects induced by the rupture of the first layer, the deformation behavior of the second layer exhibits higher apparent stiffness and reduced compliance.

The force–displacement relationship during this phase follows the same phenomenological exponential form used in Stage 1, with separate effective parameters identified for the second gel layer (Fung 1967; Gefen et al. 2003), which effectively captures the nonlinear stress–strain behavior of soft hydrogels:

6 $$F_{z,3} \left( {\mathrm{z}} \right) = {\mathrm{F}}_{{{\mathrm{z}},2}} \left( {\mathrm{z}} \right) - \frac{{\pi R^{2} }}{{\beta_{3} }}\left( {{\mathrm{e}}^{{{\upbeta}_{3} {\mathrm{k}}_{3} \left( {{\mathrm{z}} - {\mathrm{z}}_{{{\mathrm{s}}3}} } \right)/{\mathrm{R}}}} - 1} \right),$$

Where ${k}_{3}$ and ${\beta}_{3}$ are the effective stress-scale and inverse-stress nonlinearity parameters for the second gel layer, respectively, obtained by fitting the corresponding single-layer 0.7% agar gel experiments, and ${z}_{s3}$ denotes the onset position of this stage.

Stage 4: Post-penetrating insertion into the inner gel layer

After the probe punctures the interface and enters the second gel layer, as shown in Fig. 5d, the insertion force again consists of three components: (1) an exponentially decaying term describing the stress relaxation immediately following fracture, (2) a depth-dependent frictional resistance distributed along the probe surface, and (3) a constant cutting force acting at the probe tip. Owing to the higher agar concentration of the second layer, both the frictional and cutting components exhibit greater magnitudes compared with those in the outer layer, while the relaxation process occurs over a shorter characteristic length. The total insertion force in this regime is expressed as

7 $${F}_{z,4}\left(z\right)=-\left[{{A}_{4}e}^{-\frac{\left(z-{z}_{e3}\right)}{{\tau}_{4}}}+{F}_{z,fric}^{\left(4\right)}\left(z\right)+{C}_{4}\right],$$

where z is the insertion depth, ${z}_{e3}$ denotes the transition point from Stage 3, ${A}_{4}$ is the amplitude of the relaxation term, ${\tau}_{4}$ represents the relaxation length constant, ${F}_{z,fric}^{(4)}(z)$ is the frictional resistance, and ${C}_{4}$ is the cutting force. Continuity with the preceding stage is enforced through

8 $${A}_{4}={-F}_{z,3}\left({z}_{e3}\right)-{F}_{z,fric}^{\left(4\right)}\left({z}_{e3}\right)-{C}_{4},$$

ensuring a smooth transition of the force curve across the inner layer fracture boundary.

The frictional resistance is modeled as a linear function of insertion depth,

9 $${F}_{z,fric}^{\left(4\right)}\left(z\right)={\mu}_{2}\cdot 2\pi R{z}_{e2}+{\mu}_{4}\cdot 2\pi R\left(z-{z}_{e2}\right).$$

where ${z}_{e2}$ denotes the end position of Stage 2, corresponding to the length of probe insertion within the outer layer before entering the second layer. And ${\mu}_{4}$ is the effective interfacial frictional shear-stress parameter (N/mm^2^) determined from experiments using single-layer 0.7% agar gels.

#### Removal of the probe from a two-layer agar gel

After the probe reached the target depth, it was gradually withdrawn from the two-layer agar gel, as shown in Fig. 5e. During this phase, the force response is dominated by interfacial friction between the probe shaft and the surrounding gel. Unlike during insertion, no further fracture or viscoelastic relaxation occurs, and the measured force primarily reflects sliding resistance along the wetted probe–gel interface. Under the fixed withdrawal speed used in this study, the withdrawal resistance was represented by a linear embedded length-dependent friction law (Barbé et al. 2007; Abolhassani et al. 2007), where the total resistance scales proportionally with the embedded length.

To account for the different interfacial properties of the two-layer gel, the overall withdrawal force is modeled as

10 $${F}_{z,6}\left(z\right)=\left\{\begin{array}{c}2\pi R{\mu}_{6}z, (z<{z}_{s3}) \\ 2\pi R{\mu}_{6}{z}_{s3}+2\pi R{\mu}_{7}\left(z-{z}_{s3}\right), (z\ge {z}_{s3})\end{array},\right.$$

where $R=0.9\hspace{0.17em}mm$ is the probe radius, ${\mu}_{6}$ and ${\mu}_{7}$ are the effective interfacial frictional shear-stress parameter (N/mm^2^) for the outer and inner gel layers, respectively, and ${z}_{s3}$ denotes the onset of Stage 3, corresponding to the interface between the two layers. Throughout Eqs. (3)–(10), A and C have units of N, τ has units of mm, and μ has units of N/mm^2^.

#### Physics-guided residual neural network (PGNN) modeling

From the above stage-wise axial-force models, it can be observed that the probe insertion process involves multiple strongly nonlinear axial-force contributions, whereas the withdrawal process is dominated by linear axial frictional interactions. Such complexity makes it challenging for purely analytical formulations to capture all experimental variations in the measured ${F}_{z}\left(z\right)$ response and interfacial behaviors. To address this limitation and further improve axial-force prediction accuracy while preserving the stage-wise analytical structure, a stage-wise hybrid physics-guided residual neural network (PGNN) framework was developed. Physics-guided learning integrates analytical models and physics-based constraints with data-driven residual learning (Cuomo et al. 2022; Chong and Pramanik 2023). The PGNN serves as a residual correction to the analytical axial-force models described in Sects. 2.3.1 and 2.3.2, ensuring smooth transitions between stages and improved agreement with experimental ${F}_{z}\left(z\right)$ data without refitting the theoretical parameters. The total predicted axial force in each stage is represented as the sum of the analytical axial-force model and a learned residual term:

11 $${F}_{z,tot}^{(s)}(z)={F}_{z,ana}^{(s)}(z)+{s}^{(s)}(z)\hspace{0.17em}{\mathcal{N}}_{{\theta}_{s}}(z),$$

where ${F}_{z,\mathrm{a}\mathrm{n}\mathrm{a}}^{\left(s\right)}\left(z\right)$ is the stage-specific analytical axial-force model, and ${\mathcal{N}}_{{\theta}_{s}}\left(z\right)$ denotes the stage-specific residual network output for Stage s, expressed as an axial-force correction in N. The shape-weighting function ${s}^{\left(s\right)}\left(z\right)$ confines the correction to the active stage range.

Each stage-specific network ${\mathcal{N}}_{{\theta}_{s}}$ consists of four hidden layers with 64 neurons per layer and tanh activation functions. A separate network was trained for each of Stages 1–4 to account for the stage-specific force response. Each network outputs a residual axial-force correction that locally represents the unresolved discrepancy between the analytical axial-force model and the measured ${F}_{z}\left(z\right)$ response.

During training, the loss function combines data fidelity and physical regularization. The data term minimizes the difference between the predicted and measured forces using the Huber loss function, while a soft physics constraint is added in Stage 1 to limit deviations from the Fung-type nonlinear elastic law:

12 $$\mathcal{L}={\mathcal{L}}_{data}+\lambda \hspace{0.17em}{\mathcal{L}}_{phy},$$

where λ=1 for Stage 1 and λ=0 for other stages. The networks were trained using the Adam optimizer with a decaying learning rate schedule and early stopping criteria.

To maintain continuity between stages, the PGNN uses cross-stage updates based on the predicted boundary forces. Specifically, the terminal output of Stage 1 at $z={z}_{e1}$ defines the initial force ${F}_{\mathrm{m}\mathrm{a}\mathrm{x}}$ for Stage 2 (and Stage 3), while the predicted force at the end of Stage 3 determines the starting condition for Stage 4.

A limited held-out evaluation was performed using the six available cases. The network was trained on Cases 2–6 and evaluated on Case 1, which was not included during training.

#### Axial-force modeling procedure

To ensure that the stage-wise axial-force model captures the dominant mechanics of insertion and withdrawal, the modeling procedure was carried out in three sequential steps: (1) layer-specific parameter identification, (2) analytical axial-force model evaluation in the two-layer configuration, and (3) PGNN-based residual refinement.

1. Parameter identification for each gel layer

Layer-specific parameters were identified from the single-layer calibration experiments described in Sect. 2.2.1. By fitting the measured force–displacement curves to the analytical expressions in Eqs. (2)–(7), we obtained the parameter set (k₁, β₁, A₂, τ₂, C₂, μ₂) for the outer 0.6% w/w agar layer and (k₃, β₃, A₄, τ₄, C₄, μ₄) for the inner 0.7% w/w agar layer. Withdrawal tests additionally provided the effective interfacial frictional shear-stress parameters μ₆ and μ₇ for the outer and inner layers, respectively.

2) Model evaluation in the two-layer configuration

With the layer-specific parameters identified, the complete stage-wise axial-force model was applied to the two-layer agar configuration, in which the outer 0.6% w/w gel overlays the inner 0.7% w/w gel. The predicted Z-direction insertion and withdrawal forces were computed without additional fitting and compared directly with the measured ${F}_{z}\left(z\right)$ responses. This step evaluates whether the calibrated parameters transfer directly to the two-layer configuration and reproduce the measured interface transition response.

3) PGNN-based residual model refinement

Finally, the hybrid physics-guided residual neural network (PGNN) described in Sect. 2.3.3 was incorporated to provide residual corrections to the analytical axial-force predictions. The PGNN preserves the stage-wise analytical structure while learning the unresolved axial-force model discrepancy, thereby providing residual refinement without refitting the analytical parameters. Accordingly, all three modeling steps produce and evaluate the Z-direction axial force ${F}_{z}\left(z\right)$, while ${F}_{y}$, ${F}_{iy}$, and electrode deviation are retained as experimentally measured outcomes.

## Results

### Phantom material property

**Fig. 6:**
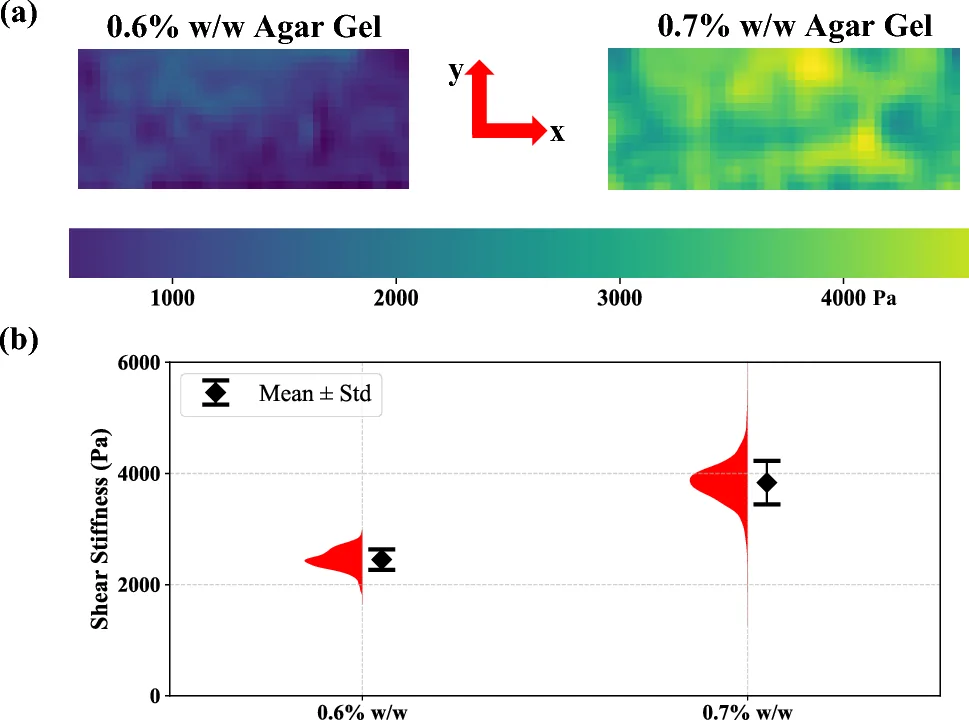
**a** Mid-slice shear stiffness maps of the 0.6% w/w and 0.7% w/w agar phantoms at 100 Hz. **b** Half-violin plots showing the voxel-wise spatial shear-stiffness distributions within the whole-phantom ROIs. Black diamonds mark the whole-phantom means, and error bars denote spatial standard deviations

The nonlinear inversion (NLI) algorithm was used to estimate the small-amplitude dynamic shear properties of agar gel phantoms from MRE displacement data (McGarry et al. 2012). NLI has been validated in phantom studies and shown to yield accurate estimates of shear modulus in comparison with mechanical testing (Solamen et al. 2018). NLI is a finite element-based iterative algorithm that solves for the spatial distribution of the complex shear modulus $G={G}^{\prime}+iG^{\prime}{\prime},$ where ${G}^{\prime}$ is the storage modulus, and ${G}^{{\prime}{\prime}}$ is the loss modulus. Shear stiffness μ was calculated from the complex modulus using the relationship $\mu = \frac{2{\left|G\right|}^{2}}{{G}{\prime}+\left|G\right|}$ (Manduca et al.^2001^) Figure 6a presents the mid-slice shear stiffness maps for agar gels at 0.6% and 0.7% w/w concentrations. The color bar represents local shear stiffness in Pascals, ranging to 5000 Pa (yellow). As expected, the higher agar concentration produced a higher recovered shear stiffness. Figure 6b shows the voxel-wise spatial distributions of shear stiffness within each whole-phantom ROI. At 100 Hz, the whole-phantom mean shear stiffness was 2.45 kPa for the 0.6% w/w gel and 3.84 kPa for the 0.7% w/w gel. Thus, the 0.1-percentage-point increase in agar concentration corresponded to an approximately 1.57-fold increase in whole-phantom mean MRE-derived shear stiffness at 100 Hz. The corresponding whole-phantom damping ratios were 0.0237 and 0.0206, respectively. Black diamonds indicate the whole-phantom means, and the error bars denote the spatial standard deviations across voxels within the corresponding ROIs. These results establish a controlled small-amplitude dynamic shear-stiffness contrast between the two agar formulations. Although the shear-stiffness magnitudes are of the same order as values reported for the human brain using MRE, the relevant literature values were obtained under different excitation conditions. This comparison, therefore, provides only contextual support for material selection and does not establish mechanical equivalence under the finite deformation associated with probe insertion**.**

### Interface characterization

**Fig. 7:**
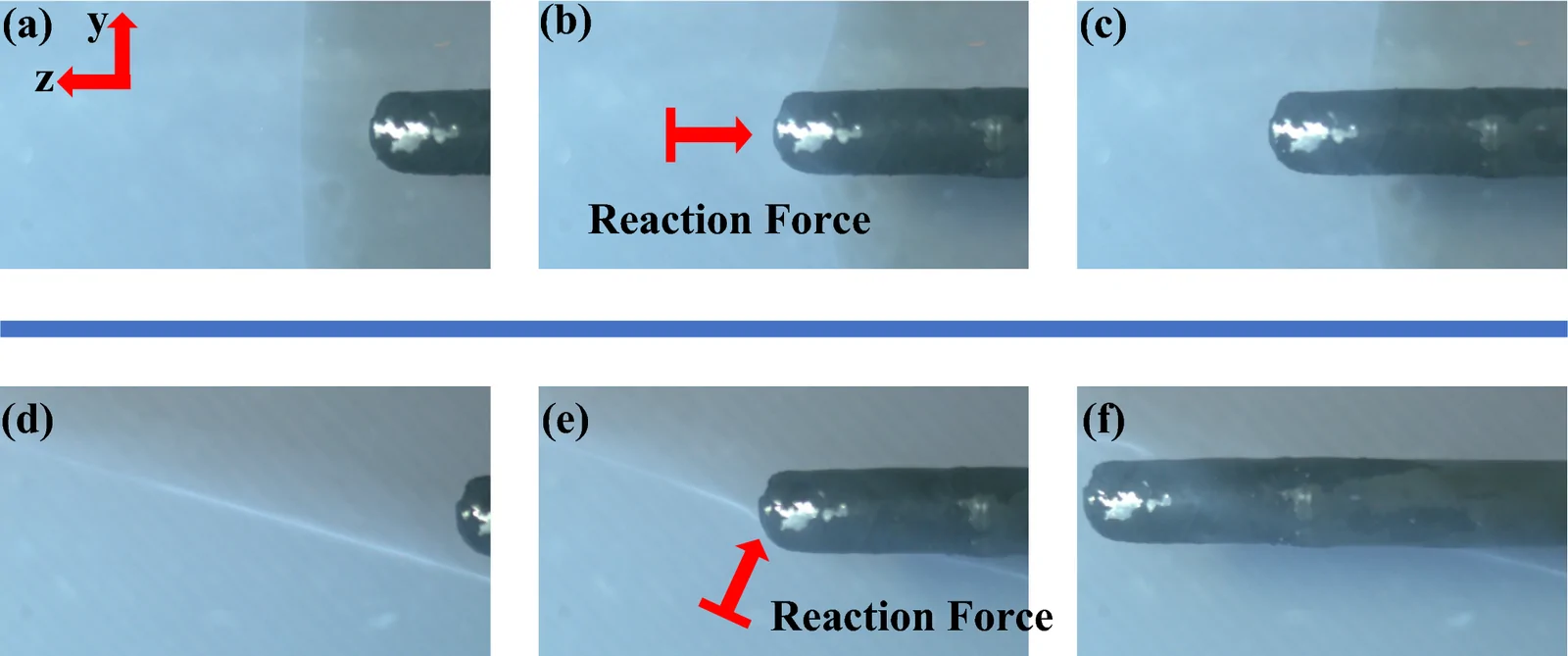
High-speed imaging of probe–gel interface deformation during insertion

Before discussing the effects of interface angle on deviation and force variations, it is essential to first characterize the deformation of the interface during the processes of probe insertion and extraction. To capture the detailed interaction as the probe crosses the interface, a Miro C110 high-speed camera (Phantom, NJ, USA) was employed to record the process at 400 frames per second (FPS). As shown in Fig. 7a–(c), when the probe is inserted perpendicularly (α = 0° in Fig. 3c) into the gel and approaches the interface, the interface deforms and generates a reaction force opposite to the probe’s motion. Once the probe fully penetrates the interface, the deformed interface relaxes, leading to a reduction in stress. Throughout this process, the interface deformation remains symmetric with respect to the probe axis.

When the interface is tilted relative to the probe (e.g., α = 75°), as illustrated in Fig. 7d–f, the penetration process becomes more complex. Due to the stiffness of the interface, a reaction force develops as the probe pushes against it; however, unlike the case at α = 0°, this deformation is not symmetric with respect to the probe’s insertion direction. Consequently, the reaction force on the interface forms an angle with the probe trajectory. At larger angles, such as 75°, the probe does not puncture the interface immediately upon initial contact but instead slides along the interface until sufficient deformation accumulates for penetration to occur. This transient sliding alters the local contact state before penetration and contributes to a more intricate deformation pattern at the interface. Once the probe completely crosses the interface, the deformed surface relaxes, leading to a decrease in the Z-direction stress. Throughout this process, the deformation of the interface remains asymmetric about the probe axis. This observed deformation asymmetry provides experimental context for the angle-dependent lateral-force and electrode deviation results reported separately in Sects. 3.3 and 3.4.

(a–c) Probe inserted perpendicularly (α = 0°): The interface deforms symmetrically and generates a reaction force opposite to the probe’s motion. (d–f) Probe inserted at a tilted interface (α = 75°): deformation becomes asymmetric, and the reaction force forms an angle relative to the probe trajectory. Videos S2 and S3, showing the corresponding experimental processes, are provided in the supplementary materials.

### Force measurement

**Fig. 8:**
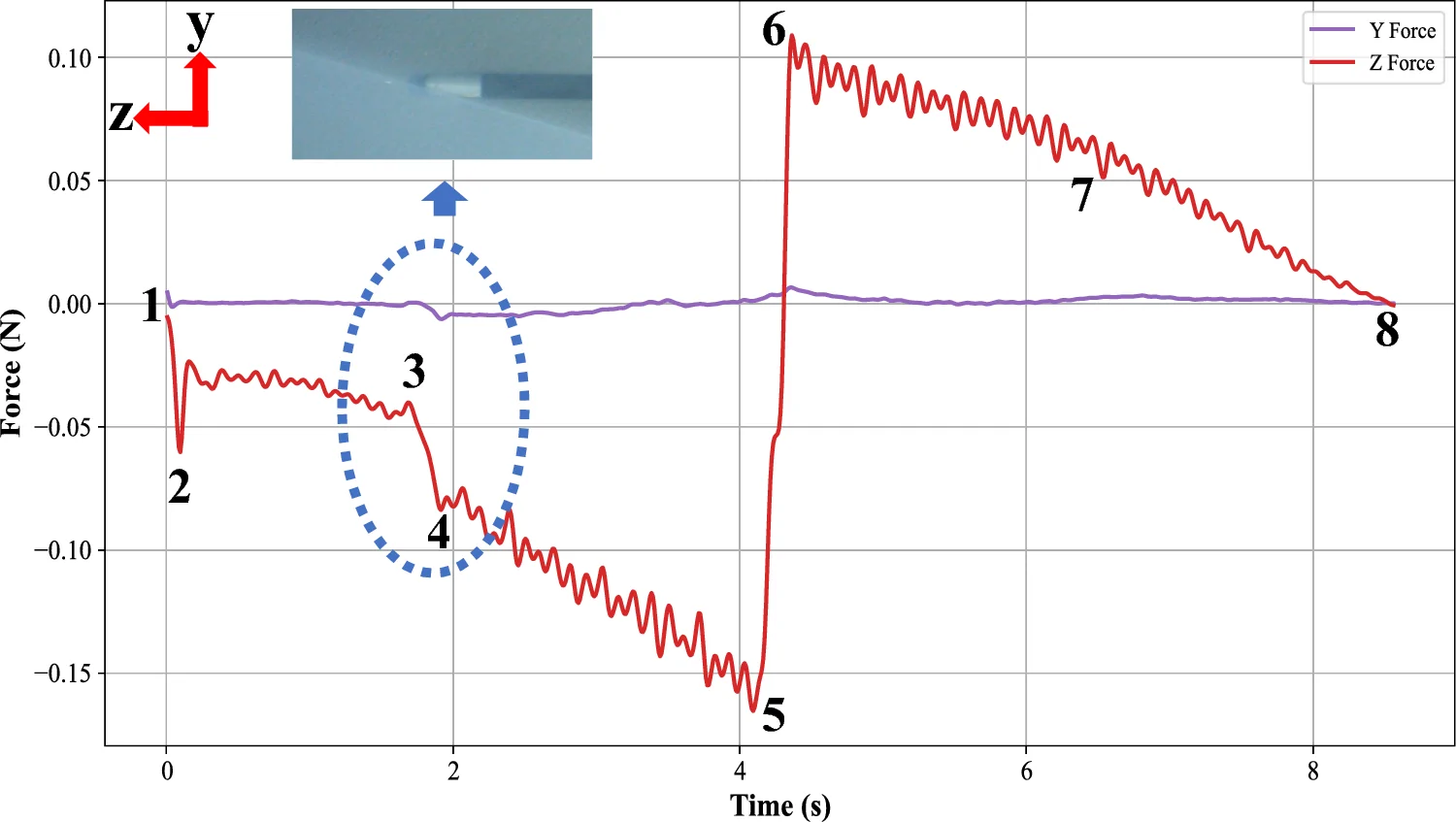
Force variations in the Y and Z directions during probe insertion and removal at a ${75}^{^\circ }$ interface angle. The highlighted region indicates the process in which the probe traverses the interface. An experiment video S4 is provided in the supplementary materials

The experimental setup depicted in Fig. 2 was used to measure forces during the insertion and removal of the probe through the dual-layer agar gel. The recorded force data, presented in Fig. 8, show distinct variations in the Z-axis and Y-axis as the probe crosses the interface between the two agar gel layers. In this setup, the Z-axis corresponds to the axial direction of the probe. The X-axis and Y-axis define the plane normal to the probe insertion, with the X-axis pointing directly toward the camera which is positioned on top of the agar gel sample. Both ${F}_{z}$ and ${F}_{y}$ were recorded directly by the force transducer. The stage-wise model was evaluated using the Z-direction ${F}_{z}\left(z\right)$ response, while the Y-direction force was analyzed independently as an experimental measurement.

For the Z-axis, as shown in Fig. 8, the probe undergoes several distinct stages. Stages 1 and 2 (points 1–3): The probe penetrates the softer layer with relatively low resistance. Stage 3 (points 3–4): As the probe reaches and deforms the interface, the Z-force decreases sharply, reflecting the additional resistance and subsequent rupture of the boundary. After puncturing the interface and entering the stiffer layer, the force response temporarily relaxes. Stage 4 (points 4–5): The reaction force rises rapidly again, dominated by the higher modulus of the harder layer. Upon reaching the maximum depth (points 5–6), the probe begins to withdraw. Withdrawal phase (points 6–8): During retraction, the Z-direction force reverses and gradually decreases toward zero.

**Fig. 9:**
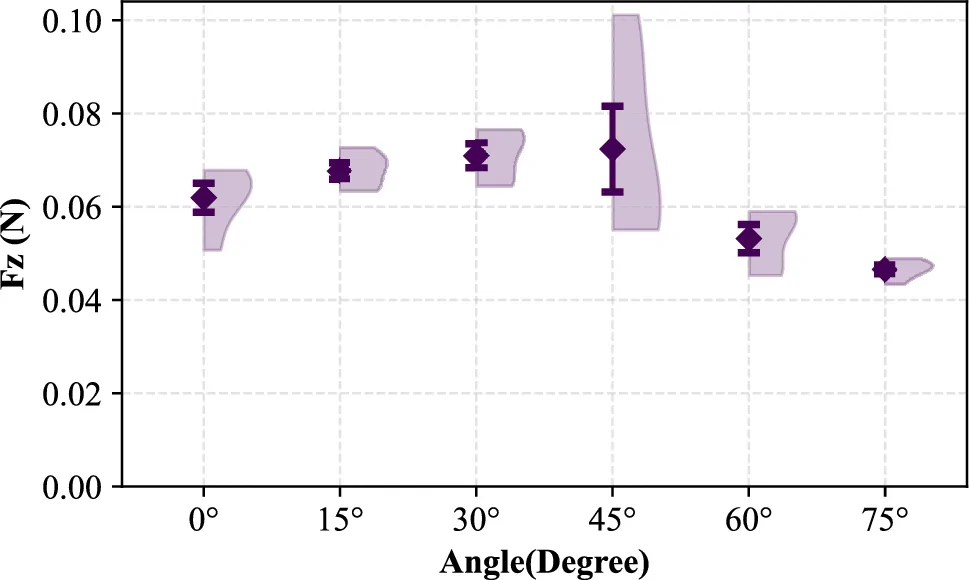
Z-axis force change at the interface (${{\boldsymbol{F}}}_{{\boldsymbol{i}}{\boldsymbol{z}}}$) variation as the probe crosses the interface, correlated with interface angle ${\boldsymbol{\alpha}}$

As illustrated in Fig. 8, a rapid force change at the interface (3–4) was defined as ${F}_{iz}$. Figure 9 and Table 1 show that ${F}_{iz}$ exhibits a weak increasing trend as the interface angle α increases up to 45°, but the relationship is not strictly monotonic: The variability is largest at 45°, and a slight reduction occurs at 60°, and a further decrease at 75°. This indicates that interface geometry affects not only the mean force change but also its variability. Statistical analysis further supports this observation: Pearson correlation between α and ${F}_{iz}$ yielded r = −0.614 with *p* = 0.195, indicating no significant linear correlation. Therefore, while descriptive statistics suggest a weak trend, the effect of interface angle on ${F}_{iz}$ is not statistically significant. The relatively large variability observed across trials suggests that axial-force response at the interface is influenced by local deformation dynamics and interface interaction conditions, rather than being governed by a single deterministic relationship with interface angle.

**Table 1:** Z-axis force change at the interface (***F***_***iz***_) and Y-axis force change at the interface (***F***_***iy***_) variation as the probe crosses the interface, correlated withinterface angle α

Angles,α		0°	15°	30°	45°	60°	75°
F_iz_	Mean(N)	0.0620	0.0677	0.0709	0.0724	0.0532	0.0466
	Std.Dev.(N)	0.00312	0.00176	0.00258	0.009198	0.00302	0.000916
F_iy_	Mean(N)	0.000886	0.00125	0.00260	0.00594	0.00689	0.00772
	Std.Dev.(N)	0.000181	0.000129	0.000189	0.00117	0.000601	0.000975

**Fig. 10:**
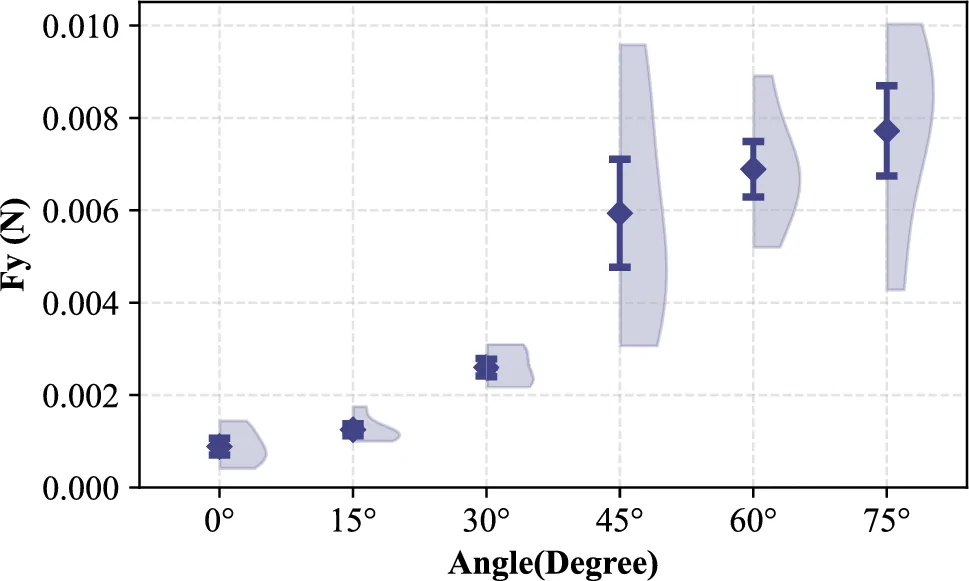
Y-axis force change at the interface (${{\boldsymbol{F}}}_{{\boldsymbol{i}}{\boldsymbol{y}}}$) variation as the probe crosses the interface, correlated with interface angle ${\boldsymbol{\alpha}}$

The lateral Y-direction force change at the interface is defined as ${F}_{iy}$. This quantity was calculated directly from the measured ${{\boldsymbol{F}}}_{{\boldsymbol{y}}}$ signal and is reported as an experimental outcome. As shown in Fig. 10 and Table 1, the mean ${F}_{iy}$ increased from 0.886 mN at 0° to 7.72 mN at 75°. The group standard deviations were generally larger for the 45°–75° conditions than for the 0°–30° conditions, but did not increase monotonically with interface angle. Pearson correlation between α and ${F}_{iy}$ yielded r=0.971 and p=0.001, indicating a significant positive association between interface angle and the group mean lateral force. This result shows that lateral force increases with interface angle and is statistically significant. At larger interface angles, the increased variability reflects more complex probe–interface interactions, in which transient sliding along the interface and asymmetric deformation prior to penetration modify the local loading condition and contribute to fluctuations in lateral force.

### Deviation measurement

**Fig. 11:**
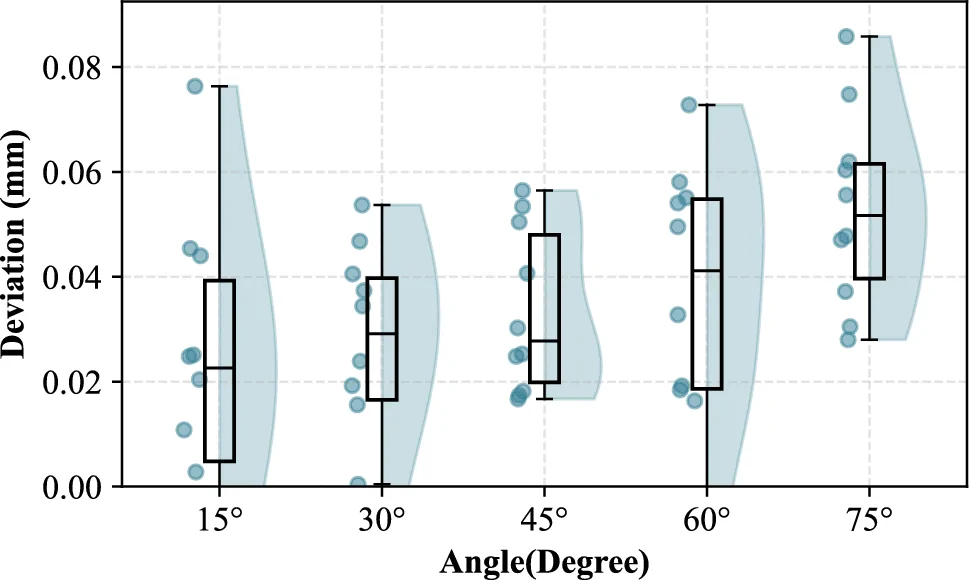
The change of electrode deviation with interface angles in the Y-direction

In actual DBS surgeries, the deviation of electrode placement from the intended position is a crucial metric for assessing surgical precision. This study primarily investigated the relationship between interface angles and electrode deviation. We observed that all deflections of the electrode occurred in the direction that increases angle β, as illustrated in Fig. 3c and d. A more detailed analysis of the electrode insertion experiments is summarized in Fig. 11 and Table 2. For comparison across interface conditions, the mean deviation of the 0° group was defined as the zero reference. The deviations reported for the 15°–75° groups, therefore, represent values relative to this baseline. Figure 11 presents the individual baseline-referenced measurements and their within-group distributions, whereas Table 2 reports the corresponding group means and standard deviations. Relative to the 0° reference, the mean deviation increased from 0.0189 mm at 15° to 0.0529 mm at 75°. The value of 0.0529 mm represents the largest group mean observed in this study rather than the maximum deviation of an individual trial. As illustrated in Fig. 11, the central tendency of the deviation distributions generally shifted upward with increasing interface angle, although considerable overlap remained between adjacent angle groups. The group standard deviations ranged from 0.0156 mm at 45° to 0.0341 mm at 15° and did not increase monotonically with interface angle. Using the five nonzero angle group means (n = 5 angle-level observations), Pearson correlation analysis yielded r=0.962 and p=0.0087, indicating a significant positive association between interface angle and group mean deviation. One-way ANOVA also indicated significant differences among the angle groups (F=2.66,p=0.045).

**Table 2:** The change of electrode deviation in the Y-direction with interface angles

Angles,α		15°	30°	45°	60°	75°
Deviation	Mean(mm)	0.0189	0.0227	0.0334	0.0357	0.0529
	Std.Dev.(mm)	0.0341	0.0285	0.0156	0.0275	0.0187

### Axial-force model results

The measured Z-direction force–displacement responses were divided into the six stages defined in Sect. 2.3, reflecting the dominant mechanics of the outer and inner gel layers throughout insertion and withdrawal. To maintain consistency with the modeling framework, the results are presented stage-by-stage, beginning with the identification of effective, experiment-specific model parameters from single-layer experiments, followed by evaluation in two-layer configurations, and concluding with PGNN-based residual refinement applied only to insertion phases.

During Stage 1 (pre-penetration of the outer layer)**,** the outer 0.6% agar exhibited a gradual, nonlinear stiffening response. This behavior was well captured by the Fung-type exponential law (Eq. 2), yielding $k_{1} = 0.0159N/mm^{2}$ and $\beta_{1} = 8.42 \times 10^{ - 4} mm/N$, consistent with the measured compliant and weakly strain-stiffening pre-puncture response.

Upon fracture, Stage 2 began, where post-penetration insertion was dominated by viscoelastic relaxation and sliding friction (Eq. 3). The corresponding relaxation length and interfacial resistance were $\tau_{2} = 1.78 mm$ and $\mu_{2} = 2.32 \times 10^{ - 4} N/mm^{2}$, along with a constant cutting term $\tau_{4} = 1.44 mm$, marking the shift from elastic resistance to more steady-state sliding.

When the probe reached the inner 0.7% layer**,** Stage 3 showed a stronger nonlinear pre-penetration response, reflected in the substantially larger $\beta_{3} = 225.0 mm/N$ and $k_{3} = 0.00719N/mm^{2}$. Because k and β enter the exponential relation as coupled parameters, their individual magnitudes should not be interpreted independently as direct measures of material stiffness or strain sensitivity.

Stage 4** (**post-fracture inner layer insertion) further exhibited a shorter relaxation constant $\tau_{4} = 1.44mm$ and higher sliding friction $\mu_{4} = 2.78 \times 10^{ - 4} N/mm^{2}$, demonstrating a more strongly resisting interfacial network.

**Fig. 12:**
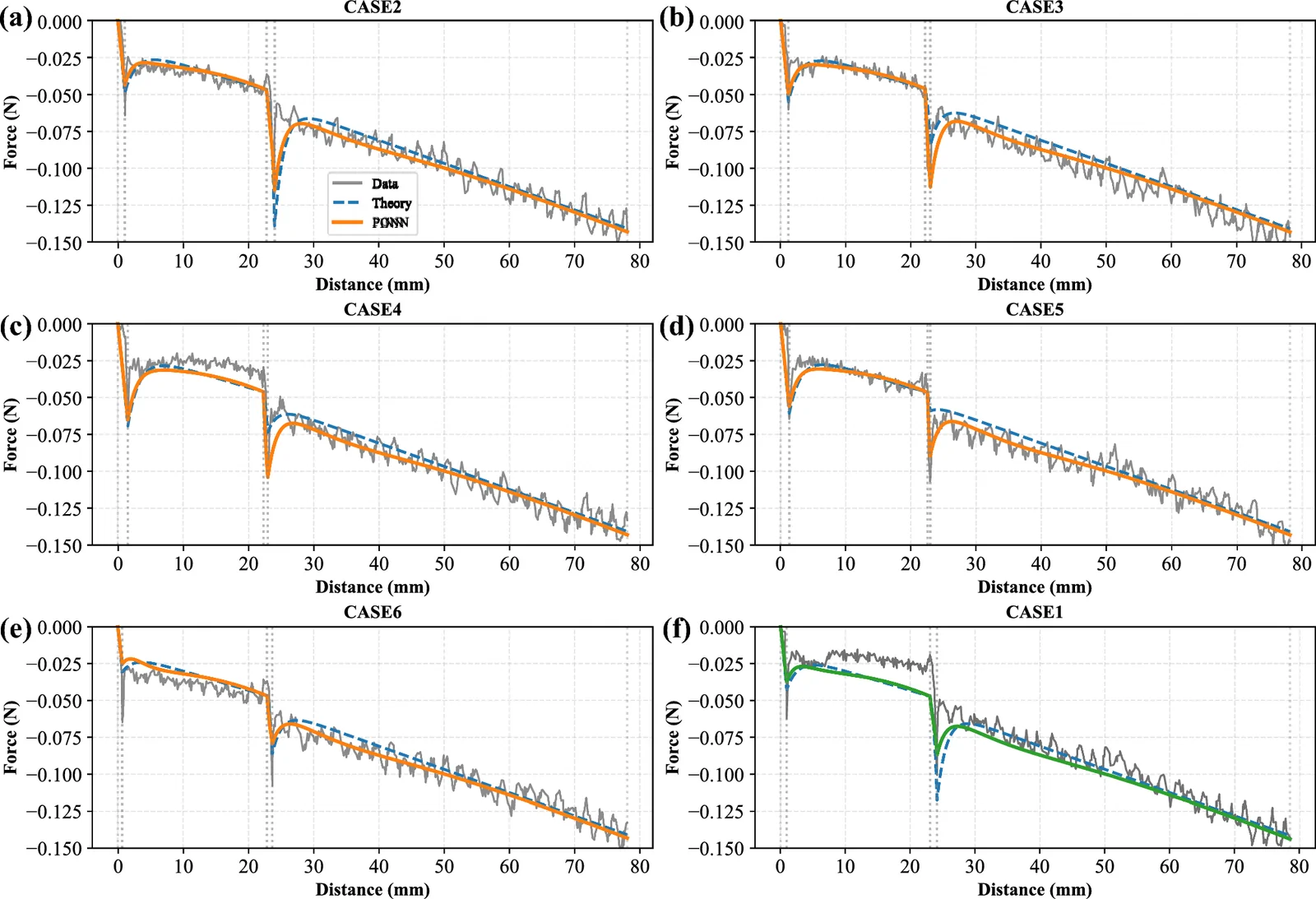
Z-direction axial force–displacement curves for six cases: experimental data (gray), analytical model (dashed), and PGNN-corrected model (solid). Panels (**a**–**e**) correspond to the five training cases (Cases 2–6), while panel (**f**) shows the held-out prediction for Case 1, which was not used during training

Using these parameters directly in the stage-wise analytical model reproduced the key features of the two-layer insertion behaviors (Fig. 12), including gradual elastic loading, abrupt fracture events, exponential force relaxation, and quasi-linear steady insertion. Minor discrepancies were most evident near the interface transitions, where sharp, localized force variations were not fully represented by the analytical relations. Across the five training cases (Cases 2–6), the analytical model achieved a mean ${R}^{2}$ of 0.938. To address the remaining discrepancies without refitting the analytical parameters, a stage-wise hybrid physics-guided residual neural network (PGNN) was explored as a residual-correction approach for Stages 1–4. As summarized in Table 3, the PGNN increased the training-case mean ${R}^{2}$ modestly from 0.938 to 0.954 $\left(\Delta {R}^{2}=0.016\right)$. The improvement was not uniform across individual cases; for Case 4, ${R}^{2}$ changed slightly from 0.947 to 0.946. For the single held-out Case 1, which was not included during training, ${R}^{2}$ increased from 0.919 for the analytical model to 0.932 for the PGNN-corrected model. These results indicate limited but measurable residual refinement within the present five-training-case/one-held-out-case evaluation.

**Table 3:** R^2^ values for the five training cases (Cases 2–6) and the single held-out test case (Case 1), comparing the analytical and PGNN-corrected axial-force models

Case number	Analytical model R^2^	PGNN model
Training case		
Case 2	0.913	0.946
Case 3	0.943	0.961
Case 4	0.947	0.946
Case 5	0.942	0.964
Case 6	0.946	0.953
Test case		
Case 1	0.919	0.932

**Fig. 13:**
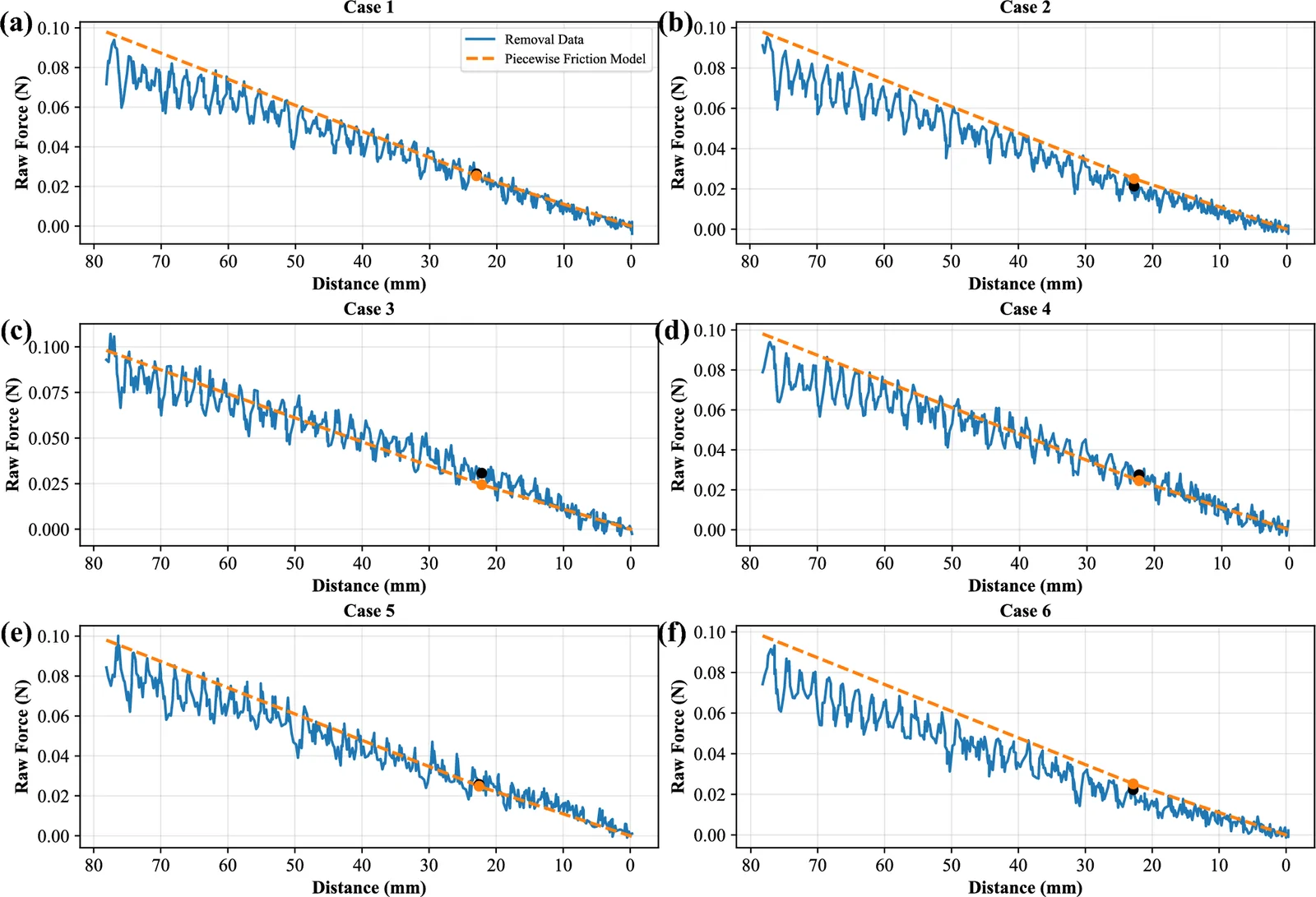
Comparison between experimental removal forces and modeled Z-direction axial withdrawal forces for two-layer gels (Cases 1–6)

Once insertion was completed, the withdrawal process (Stage 6) required no additional fitting. The probe traveled along a pre-fractured path, and the removal force was governed entirely by interfacial sliding friction, with ${\mu}_{6}\approx {\mu}_{2}$ for the outer layer and ${\mu}_{7}\approx {\mu}_{4}$ for the inner layer. Across six two-layer withdrawal experiments, this friction-only law achieved $R^{2} = 0.704{-}0.923$ (mean 0.838) and reproduced the linear scaling of force with embedded length (Fig. 13), confirming that sliding dominates removal mechanics and that friction parameters identified from single-layer gels transfer directly to two-layer conditions.

## Discussion

### Experimental findings in two-layer phantoms

The experimental results show that interface angle was positively associated with both the directly measured lateral interface-force change and the independently measured electrode deviation. High-speed imaging documented asymmetric deformation and transient sliding at inclined interfaces, providing qualitative mechanical context for these angle-dependent trends. Together, these observations establish interface orientation as a controlled factor shaping lateral loading and electrode deviation in the phantom system and motivate future paired measurements to quantify trial-level force–trajectory coupling. Our recent study characterized probe insertion forces and probe created channel deformation in a homogeneous agar phantom (Chen et al. 2026). The present study extends that controlled platform by introducing a prescribed, MRE-characterized stiffness contrast across a planar interface and systematically varying the interface orientation. Specifically, our phantom reproduces a simplified insertion scenario inspired by a clinical DBS approach in which an RF probe with dynamic impedance monitoring is used to establish a low-resistance channel prior to electrode insertion (Fayed et al. 2024). By reconstructing this procedure in a controlled phantom setting, we were able to isolate the effect of interface angle while minimizing confounding factors such as brain shift, physiological motion, and multiple simultaneous boundaries.

The two-layer phantom design created a prescribed contrast between a lower-stiffness 0.6% agar layer and a higher-stiffness 0.7% agar layer. MRE at 100 Hz characterized this contrast under small-amplitude dynamic loading and provided frequency-specific mechanical context for material selection. For physiological context, Hiscox et al. (2020) reported mean MRE-derived shear stiffness values at 50 Hz of 2.37 kPa for cortical gray matter, 2.95 kPa for white matter, and 3.46 kPa for subcortical gray matter. These values correspond to regional contrasts of approximately 1.24 for white matter and 1.46 for subcortical gray matter relative to cortical gray matter. The 1.57-fold contrast in the present phantom is, therefore, of a comparable order of magnitude, although differences in material type and excitation frequency preclude direct tissue matching. Despite this contextual similarity, the phantom MRE measurements do not establish equivalence to cortical or subcortical brain tissue under the finite deformation, rupture, and frictional contact associated with probe insertion. The controlled stiffness contrast nevertheless enabled systematic examination of how interface angle influenced the measured probe–interface forces and, in a separate experimental series, electrode deviation.

High-speed imaging showed that inclined interfaces can introduce asymmetric deformation and transient sliding before penetration, creating a more complex local contact pathway. Consistent with this observation, the lateral-force measurements showed broader within-group distributions for the 45°–75° conditions than for the 0°–30° conditions, although the standard deviation did not increase monotonically with angle. The electrode deviation standard deviations likewise showed no monotonic angle dependence. These distributions indicate that the angle-dependent changes in group means and the within-group variability represent distinct features of the probe–interface response and should be interpreted separately.

An important limitation is that MRE characterized the small-amplitude dynamic response of the agar gels at 100 Hz, whereas probe insertion involves finite deformation, rupture, and frictional contact. Independent large deformation compression, shear, or indentation tests were not performed to establish quantitative equivalence between these agar formulations and brain tissue. Moreover, the measured damping ratios were substantially lower than values reported for brain tissue, consistent with the known limitations of agar phantoms as viscoelastic tissue surrogates (McIlvain et al. 2019). The present two-layer phantom contains a single planar interface and a single prescribed stiffness contrast; it does not reproduce continuous stiffness gradients, anisotropy, fluid-related effects, physiological motion, or the broader heterogeneity of the brain. For a curved anatomical interface, n would be defined from the local tangent plane at the probe–interface contact point, and curvature could cause α to vary along the insertion path. The present analysis also considers only the in-plane Y-direction lateral-force component and does not resolve non-coplanar three-dimensional lateral loading. Insertion speed was fixed within each experimental series, enabling the angle-dependent response to be isolated at the tested speed; consequently, the present study does not characterize angle–velocity interactions. Future work will extend the electrode deviation experiments to multiple insertion speeds. Accordingly, the absolute insertion forces, fitted model parameters, and electrode deviations reported here should not be transferred directly to the human brain. The phantom and modeling framework are intended as a controlled mechanistic platform for isolating the relative effect of interface orientation under a prescribed stiffness contrast.

### Analytical and physics-guided residual model interpretation

To quantitatively interpret the force–displacement behavior across the entire insertion–withdrawal cycle, we developed a six-stage analytical model based on nonlinear indentation, interface fracture, viscoelastic relaxation, and frictional sliding. The fitted parameters corresponded to specific terms in the analytical formulation: (k,β) governed the phenomenological nonlinear pre-puncture response**,** τ represented the post-fracture relaxation length**,** C represented the constant tip cutting force**,** and μ represented the effective interfacial frictional shear-stress parameter. A key observation from the modeling is that the mechanical contrast between layers appears more pronounced after fracture than before, although this interpretation is based on model fitting and requires further validation. While the inner layer showed only a moderate increase in pre-penetration stiffness, it exhibited substantially higher interfacial friction (μ₄ > μ₂) and shorter relaxation length (τ₄ < τ₂). This pattern indicates that the fitted axial-force terms differ between the two gel layers; establishing how these differences relate to electrode deviation will require paired lateral-force and trajectory measurements.

Building upon the analytical model, a stage-wise PGNN was explored as a secondary residual correction approach. Across the five training cases, the mean ${R}^{2}$ increased modestly from 0.938 to 0.954, while the single held-out Case 1 improved from 0.919 to 0.932. The PGNN, therefore, provided limited but measurable residual refinement rather than a substantial improvement in overall predictive accuracy. This refinement is interpreted as a reduction in unresolved axial-force model discrepancy rather than as evidence for a specific viscoelastic or heterogeneous mechanism. The angle–lateral-force and angle–deviation relationships were established directly from their corresponding experimental measurements and are presented as complementary findings alongside the axial model results.

Experimentally, the group mean lateral interface-force change ${F}_{iy}$ increased significantly with interface angle, whereas the axial interface-force change ${F}_{iz}$ showed no statistically significant linear association with angle. In the separate electrode deviation experiments, the baseline-referenced group mean deviation also increased significantly with interface angle. These two datasets, therefore, reveal complementary angle-dependent trends at the group level. In addition, all measured deviations were directed toward increasing angle β, indicating a consistent directional bias under the tested interface geometry. Future experiments combining paired force and trajectory measurements will enable the trial-level coupling between lateral loading and electrode deviation to be quantified.

From a mechanistic standpoint, these findings identify interface orientation and stiffness contrast as potential contributors to trajectory deviation under controlled phantom conditions. Although clinically significant electrode deviations have been reported in DBS surgery (Philipp et al. 2021), the largest group mean deviation measured here was 0.0529 mm at 75° relative to the zero-referenced 0° group and is unlikely to be clinically significant in isolation. However, a clinical DBS trajectory traverses multiple stiffness interfaces. When successive interface interactions bias the insertion path in the same direction, their contributions are expected to accumulate and may produce a substantially larger target deviation. This interpretation is consistent with human evidence showing that a larger approach angle to an MRE-defined stiffness interface is associated with greater electrode deviation (Wu et al. 2025) and that patient-specific MRE-derived brain mechanical properties are important contributors to target deviation (Zhou et al. 2026). Depending on interface orientation, stiffness contrast, curvature, and the evolving probe-created channel, some contributions may also partially cancel or combine nonlinearly. The present study, therefore, demonstrates and quantifies an elementary single-interface response that may contribute to the more complex deviations observed clinically and provides a basis for future multi-interface and biological tissue studies.

## Conclusions

This study provides an integrated experimental–computational investigation of probe insertion mechanics in a two-layer soft material system under controlled phantom conditions. By reconstructing a simplified insertion procedure in a two-layer agar phantom with a controlled stiffness contrast characterized by MRE at 100 Hz, we were able to isolate and quantify the mechanical role of a prescribed layer interface during probe advancement and withdrawal.

The first contribution of this work lies in the development of a stage-wise axial-force model that characterizes the insertion process in terms of nonlinear indentation, fracture, post-rupture relaxation, and frictional sliding. The model parameters identified from single-layer experiments retained explicit, experiment-specific model definitions and, when transferred directly to the two-layer setting, reproduced the major features of the measured Z-direction axial force–displacement responses. The subsequent stage-wise PGNN provided modest axial-force residual refinement without refitting the analytical parameters. The PGNN, therefore, serves as an exploratory extension that quantifies the scope of residual refinement while preserving the stage-wise analytical structure.

The second contribution is the experimental demonstration that interface angle was positively associated with both the directly measured lateral interface-force change and the independently measured, baseline-referenced electrode deviation. The mean ${F}_{iy}$ increased from 0.886 mN at 0° to 7.72 mN at 75°, while the largest group mean deviation was 0.0529 mm at 75°. These complementary datasets identify interface orientation as a controlled factor shaping lateral loading and electrode trajectory under the investigated phantom conditions. Future paired force–trajectory measurements, combined with three-dimensional contact–bending models, will enable the quantitative coupling between lateral loading and electrode deviation to be established.

Together, these findings demonstrate under controlled phantom conditions that mechanical heterogeneity and interface geometry can influence probe–interface forces and electrode trajectory. The stage-wise axial-force framework and the complementary lateral-force and electrode deviation measurements provide a basis for future studies incorporating independently characterized large-deformation tissue properties, anisotropy, multiple interfaces, and time-dependent deformation. Although the measured single-interface deviation is unlikely to be clinically significant in isolation, contributions from multiple interfaces are expected to accumulate when they act in the same direction along a clinical trajectory. Validation in biological tissues and anatomically complex geometries will be necessary to quantify this cumulative response and determine its relevance to DBS trajectory planning.

## Supplementary Information

Below is the link to the electronic supplementary material.

Supplementary file1 (MP4 233 KB)

Supplementary file2 (MP4 13194 KB)

Supplementary file3 (MP4 12887 KB)

Supplementary file4 (MP4 13727 KB)

## Data Availability

The data that support the findings of this study are available from the corresponding author upon reasonable request.
